# Structure and Absolute Configuration of 20β-Hydroxyprednisolone, a Biotransformed Product of Predinisolone by the Marine Endophytic Fungus *Penicilium lapidosum*

**DOI:** 10.3390/molecules190913775

**Published:** 2014-09-03

**Authors:** Sadia Sultan, Muhammad Zaimi bin Mohd Noor, El Hassane Anouar, Syed Adnan Ali Shah, Fatimah Salim, Rohani Rahim, Zuhra Bashir Khalifa Al Trabolsy, Jean-Frédéric Faizal Weber

**Affiliations:** 1 Faculty of Pharmacy, Universiti Teknologi MARA, Puncak Alam Campus, 42300 Bandar Puncak Alam, Selangor Darul Ehsan, Malaysia; 2 Atta-ur-Rahman Institute for Natural Products Discovery (AuRIns), Universiti Teknologi MARA, Puncak Alam Campus, 42300 Bandar Puncak Alam, Selangor Darul Ehsan, Malaysia

**Keywords:** 20β-hydroxyprednisolone, endophytes, *Penicilium lapidosum*, DFT, TD-DFT, ECD spectra

## Abstract

The anti-inflammatory drug predinisolone (**1**) was reduced to 20β-hydroxyprednisolone (**2**) by the marine endophytic fungus *Penicilium lapidosum* isolated from an alga. The structural elucidation of **2** was achieved by 1D- and 2D-NMR, MS, IR data. Although, **2** is a known compound previously obtained through microbial transformation, the data provided failed to prove the C20 stereochemistry. To solve this issue, DFT and TD-DFT calculations have been carried out at the B3LYP/6–31+G (d,p) level of theory in gas and solvent phase. The absolute configuration of C20 was eventually assigned by combining experimental and calculated electronic circular dichroism spectra and ^3^J_HH_ chemical coupling constants.

## 1. Introduction

Endophytes are microorganisms, *i.e.*, fungi and bacteria, defined by their ecological niche. They reside within the plant tissues beneath the epidermal cell layers and leave the host tissues symptomless, at least transiently [1]. They have often mutualistic relations with their hosts, protecting plants against herbivores, insect attacks, or tissue-invading pathogens [2]. Natural product chemists, always looking for organisms with special adaptations, are paying more and more attention to endophyte chemistry and metabolism. Indeed, a variety of bioactive substances such as paclitaxel [3], lasiodiplodins [4], and polyesters [5] have been isolated in recent years from endophytes. Various enzymes (e.g., cellulase, protease) produced by endophytes (e.g*.*, *Penicillium* sp., *Fusarium graminearum*, *Lasidiplodia* sp.) have been characterized [6,7]. Thus, endophytic fungi could become interesting tools for bioconverting exogenous compounds within the wider framework of green chemistry. 

**Figure 1:**
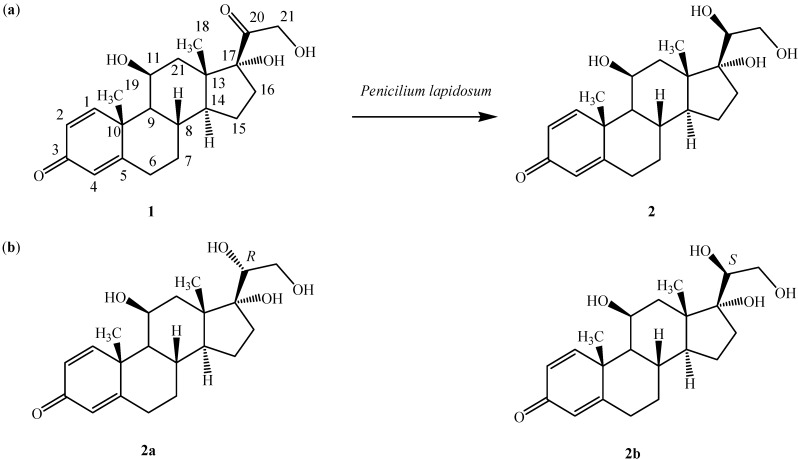
(**a**) Biotransformation of prednisolone (**1**) to 20β-hydroxyprednisolone (**2**) by *Penicilium lapidosum*; (**b**) molecular structures of stereoisomers **2a** and **2b**.

In the course of our work on microbial transformations [8,9,10,11,12,13], we have studied the transformation of various bioactive steroids such as mesterolone, hydrocortisone and tibolone [14,15,16]. In the present study we have chosen the anti-inflammatory drug predinisolone (11β,17α,21-trihydroxypregna-1,4-diene-3,20-dione, **1**, Figure 1a), as a model compound and investigated its metabolism by marine endophytes. 

Little work has been done with this type of steroids. The green alga *Scenedesmus quadricauda* T76 was cultured with prednisolone and yielded two transformed products, including a 5α-hydroxy derivative by simple hydration of the Δ^4^ double bond and a pentacyclic rearrangement product resulting from an acid enolization of the C3 carbonyl [17]. In a different study, *Acremonium strictum* was found to transform **1** by alteration of the side chain of the substrate into two steroid derivatives, which were 21,21-dimethoxy-11β-hydroxypregn-1,4-dien-3,20-dione and 11β-hydroxyandrost-1,4-dien-3,17-dione [18]. Prednisolone (**1**) was claimed to be converted into 20β-hydroxyprednisolone by *Streptomyces roseochromogenes* TS79 [19], although it should be noted that no spectroscopic evidence for the stereochemistry at C20 was provided. A similar biotransformation was observed as a minor product with dominant 16α-hydroxylation when hydrocortisone was fermented with the same bacterium [20]. 

Herein, we report the stereoselective transformation of **1** into 20β-hydroxyprednisolone (**2**) by the marine endophyte isolate SSW identified as *Penicilium lapidosum*. The absolute configuration at C20 was confirmed by predicting the spectroscopic data of stereoisomers **2a** and **2b** using DFT and TD-DFT methods and comparing them with the actual analytical data.

## 2. Results and Discussion

Endophyte SSW was isolated from an unidentified green alga collected on the Malaysian West coast. When grown on PDA, it was initially white and later turned extensively dark green. Sporulation was observed after 2 weeks of incubation. A combination of morphological and molecular analyses led to its identification as *Penicilium lapidosum*. This fungus was included in a series of 20 unidentified fungi from our collection and ATCC strains for primary screening. Only SSW provided encouraging results. As a result, large-scale fermentation was undertaken which led to the isolation of compound **2** believed to be a biotransformed product. 

Compound **2** was obtained as a white amorphous solid (5.5 mg). It was shown to have the molecular formula C_21_H_30_O_5_ from its ESI-ToF-MS ion peak observed at *m/z* 362.2134 [M]^+^ (calcd. 362.2143), or two units higher than that of the substrate **1**, indicating a reduction of a double bond. The ^1^H-NMR spectrum of **2** differed from that of **1** only by the additional resonance of H_2_O appearing at δ 3.80. The ^13^C-RNMR showed a signal at δ 75.02 ascribed to the newly formed hydroxymethine group instead of prednisolone’s C = O at δ 216.21. The rest of the chemical shifts were identical to those of prednisolone. Thus, compound **2** was identified as 20-hydroxyprednisolone. Despite an extensive literature search, no clear evidence could be used to establish the stereochemistry of C20. Therefore, computational methods were used to tackle this issue. A conformational study was carried out to identify the stable conformers of diastereoisomers **2a** and **2b**. This was followed by prediction of UV/vis, ECD and NMR data for both stereoisomers. These calculations were performed using density functional theory (DFT) and time-dependent DFT (TD-DFT) methods at the B3LYP/6–31G(d) level of theory.

### 2.1. Conformational Study

Stable conformers for **2a** and **2b** were optimized using DFT, and the minima were confirmed by the absence of imaginary frequencies. The most stable conformer for each stereoisomer **2a** and **2b** represented 63% and 66%, respectively. However, the steroid nucleus is known for its rigidity and the remaining conformers merely accounted for the flexible side chain. Therefore, all calculations were performed considering the most stable conformers for **2a** and **2b**. Incidentally, conformer **2a** was found to be more stable than **2b** by 5 kcal/mol.

### 2.2. ECD and UV/Vis Spectra Prediction

UV/Vis spectra of **2a** and **2b** stereoisomers were predicted using the TD-DFT method. The calculated maximum absorption bands (λ_MAX_) of the two stereoisomers are shown in Table 1. These bands correspond to an electronic transition from HOMO to LUMO orbitals. Solvent effects were taken into account implicitly by using the polarizable continuum model (PCM) [21]. When compared to data obtained in gas phase, the presence of PCM solvent (CH_3_CN) induces a bathochromic shift as well as an hyperchromic effect on the intensity of λ_MAX_ as seen from the values of the oscillator strength (*f*). As shown in Table 1, the calculated λ_MAX_ in PCM solvent are in good agreement with the experimental value (Δλ = 1 nm). Not surprisingly, both stereoisomers are predicted to give rise to the same UV/vis spectrum as the chromophore is as distant as possible from C20.

**Table 1:** Calculated λ_MAX_ (nm), E_MAX_ (eV) and *f* for **2a** and **2b** stereoisomers obtained at the B3LYP/6–31+G(d,p) level of theory.

	Gas			PCM			Experimental	
	λ_MAX_	E_MAX_	*f*	λ_MAX_	E_MAX_	*f*	λ_MAX_	E_MAX_
**2a**	231	5.37	0.12	239	5.19	0.25	240	4.18
**2b**	231	5.36	0.11	239	5.19	0.25		

**Figure 2:**
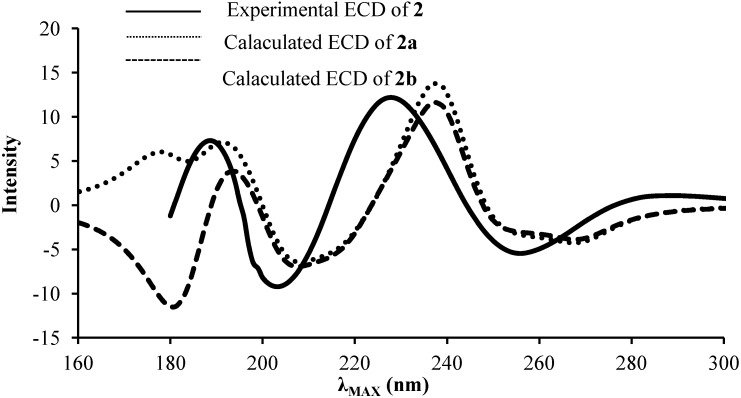
Experimental and predicted ECD spectra of **2a** and **2b** stereoisomers obtained at the B3LYP/6–31+G(d,p) level of theory.

In an attempt to determine the stereochemistry at C20 of compound **2**, ECD spectra of both stereoisomers **2a** and **2b** were predicted using TD-DFT (Figure 2) and compared to the experimental ECD of compound **2**. The predicted spectra for **2a** and **2b** showed similar trends in 190–300 nm region. However, at the region below 190 nm, the Cotton effect for the two stereoisomers showed distinct differences, which are assigned to the asymmetric C20 carbon. The experimental ECD of **2** displays between 180 and 190 nm a negative slope more likely corresponding to stereoisomer **2b**. Therefore, the absolute configuration of C20 was suggested to be *S* and thus **2** to be 20β-hydroxypredinisolone.

### 2.3. NMR Spectra Prediction

The experimental and predicted ^1^H- and ^13^C-NMR chemical shifts of stereoisomers **2a** and **2b** of compound **2** are reported in Table 2 and Table 3.

**Table 2:** Experimental and predicted ^1^H-NMR chemical shifts (δ ppm) of stereoisomers **2a** and **2b** obtained at the B3LYP/6–31+G(d,p) level of theory.

	2a		2b		Experimental
	Gas	PCM	Gas	PCM	
H1	6.85	7.45	7.01	7.52	7.5
H2	6.24	6.11	6.33	6.17	6.25
H4	5.97	5.86	6.06	5.91	6.01
H6a	2.10	2.23	2.08	2.22	2.34
H6b	2.49	2.70	2.47	2.70	2.36
H7a	1.15	1.19	1.13	1.18	1.12
H7b	1.96	2.07	1.92	2.05	2.12
H8	2.28	2.33	2.28	2.34	2.16
H9	1.08	1.14	1.08	1.13	1.11
H11	4.45	4.37	4.49	4.37	4.38
H12a	1.78	1.44	2.12	1.91	1.57
H12b	2.03	2.02	2.49	2.12	2.06
H14	2.02	1.85	2.00	1.85	1.77
H15a	1.48	1.52	1.45	1.51	1.51
H15b	1.64	1.74	1.61	1.74	1.8
H16a	0.59	0.99	0.71	1.07	1.54
H16b	3.16	2.77	1.95	2.01	2.54
H18	1.11	1.01	1.37	1.09	1.15
H19	2.15	1.95	2.13	1.95	1.52
H20	4.07	3.88	3.97	3.87	3.8
H21a	3.28	3.38	3.54	3.52	3.61
H21b	4.02	3.91	3.64	3.68	3.64

**Table 3:** Experimental and predicted ^13^C-NMR chemical shifts (δ ppm) of stereoisomers **2a** and **2b** obtained at the B3LYP/6–31+G(d,p) level of theory.

	2a		2b		Experimental
	Gas	PCM	Gas	PCM	
C1	154.2	159.2	154.4	159.2	159.11
C2	130.6	125.1	130.1	124.8	126.28
C3	185.2	184.6	185.3	184.7	187.76
C4	124.1	119.7	123.9	119.4	120.94
C5	172.2	176.6	172.0	176.7	173.88
C6	32.6	33.2	31.8	32.3	31.9
C7	35.3	36.0	34.5	35.2	33.7
C8	32.2	33.2	31.5	32.6	31.2
C9	60.9	58.5	59.8	57.6	55.6
C10	46.5	49.6	45.5	48.8	44.2
C11	75.0	74.4	74.5	73.9	69.9
C12	36.9	39.9	37.6	41.2	47.3
C13	51.4	50.8	52.1	51.2	46.9
C14	52.3	51.6	51.8	51.1	56.9
C15	23.4	24.3	23.2	24.1	23.9
C16	32.0	31.0	37.3	35.9	35.2
C17	88.2	86.7	86.4	85.3	84.94
C18	14.9	16.0	12.9	14.1	16.42
C19	20.9	19.7	20.0	18.7	21.3
C20	72.3	71.6	76.9	75.8	75.02
C21	65.3	64.6	64.7	63.9	63.98

**Figure 3:**
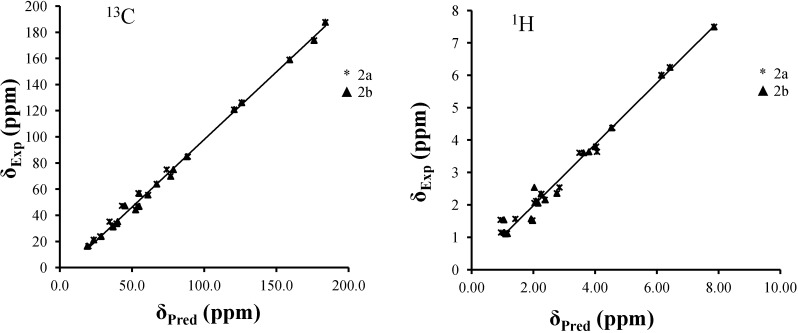
Correlation curves between predicted and experimental chemical shifts of stereoisomers **2a** and **2b** in PCM.

The correlation curves between the experimental and predicted chemical shifts of **2a** and **2b** stereoisomers are displayed in Figure 3. For both ^1^H- and ^13^C-NMR predictions, the best reproductions were obtained with the stereoisomer **2b**. For instance, the correlation coefficients obtained for ^1^H and ^13^C-NMR prediction of **2a** and **2b** in gas phase are 96.24% and 95.89%, and 99.50% and 99.42%, respectively. The small yet possibly significant differences between the correlation coefficients (1% and 3.5% for ^13^C and ^1^H-NMR correlations, respectively) could be due to the difference in stereochemistry at C20. Taking into account the solvent (PCM) used to compute solute-solvent interactions, this gives rise to only slightly better correlations for ^13^C chemical shift prediction compared to the gas phase (e.g., 99.42% and 99.58% for of **2b** in gas and PCM, respectively). Differences are larger when it comes to ^1^H chemical shift prediction, as protons are well known to be more sensitive to solvent effects than ^13^C (e.g., 95.89% and 98.55% for of **2b** in gas and PCM, respectively). However, the PCM does not really consider H-bonding interactions between solute and solvent, which significantly affect ^1^H chemical shifts. The key results here are the predicted chemical shifts in both gas and PCM of C20, which are much closer for **2b** than **2a** to the experimental value. 

**Figure 4:**
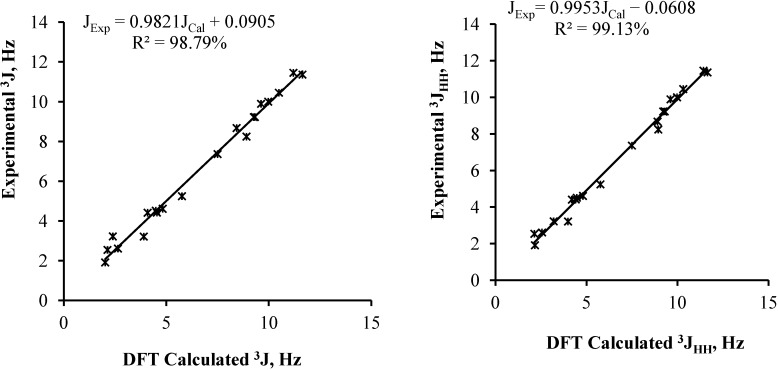
Correlation curves between experimental and PCM predicted ^3^J_HH_ coupling constants of **2a** and **2b**.

To further confirm the absolute configuration of C20, ^3^J_HH_ coupling constants were calculated for stereoisomers **2a** and **2b** at the same level of theory (Table 4). The linear correlations between the experimental coupling constants ^3^J_HH_ of compound **2** and the predicted one of stereoisomers **2a** and **2b** are shown in Figure 4. Here also, a slightly better correlation was obtained with stereoisomer **2b** (R^2^ = 99.13%) compared to **2a** (R^2^ = 98.97%). However, very significant data were obtained from ^3^J_20–21_, which for **2b** were calculated as 3.19 and 8.90 Hz. This corresponds to a variation of 0.03 and 0.27 Hz to the experimental values, respectively, which is below the experimental resolution in standard conditions. Meanwhile, small negative values were obtained for isomer **2a**. 

**Table 4:** Experimental and PCM calculated ^3^J_HH_ coupling constants for **2a** and **2b**.

	2a	2b	Experimental
H1-H2	9.97	9.97	10.0
H6-H7	5.76	5.76	5.24
	11.64	11.64	11.36
	2.13	2.13	2.54
	4.82	4.80	4.61
H7-H8	4.49	4.46	4.51
	9.62	9.61	9.89
H8-H9	9.32	9.29	9.23
H9-H11	4.09	4.19	4.41
H11-H12	2.64	2.55	2.61
	3.90	3.98	3.21
H8-H14	8.91	8.94	8.24
H14-H15	10.49	10.31	10.45
	7.50	7.49	7.36
H15-H16	4.54	4.40	4.42
	11.20	11.42	11.45
	9.27	9.21	9.22
	2.02	2.16	1.91
H20-H21	−0.82	3.19	3.22
	−1.84	8.90	8.67

## 3. Experimental Section

### 3.1. General Information

Predinisolone (**1**) was purchased from Sigma-Aldrich (St. Louis, MI, USA). Melting points were determined on a Yanaco MP-S3 apparatus (Kyoto, Japan). UV/vis spectra in acetonitrile were measured on a Shimadzu UV 240 spectrophotometer (Kyoto, Japan). A Jasco DIP-360 digital polarimeter (Gross-Umstadt, Germany) was used to measure the optical rotations in chloroform using a 10-cm cell tube, while CD spectra were acquired on a Jasco J-815 spectrophotometer in the range 200–350 nm employing a 1-cm quartz cell. A Bruker Avance III 600 Ascend spectrometer (Billerica, MA, USA) was used to record ^1^H-NMR and 2D NMR spectra at 600 MHz and ^13^C-RNMR spectra at 150 MHz using CDCl_3_ as solvent. Chemical shifts were reported in δ (ppm), relative to Si(CH_3_)_4_ as internal standard, and coupling constants (J) were measured in Hz. HR-ESI-MS data were measured on an Agilent 6224 TOF-LC/MS (Santa Clara, CA, USA), while EI-MS data were obtained from a Jeol HX 110 mass spectrometer (Tokyo, Japan). HPLC separations were performed using an Agilent HPLC system that includes a vacuum degasser G1379B, binary pump SL G1312B, autosampler G1329A, multiwavelength detector G1315B set at 220 nm, and HPLC Chemstation^®^ software B.031 SR1. Analytical scale experiments were carried on a Synergi™ Hydro-RP 80Å column (250 × 4.6 mm, 4 µm particle size, Phenomenex^®^, Torrance, CA, USA), while purification at semi-preparative scale employed a similar column with an ID of 10 mm. All reagents used were of analytical grade. 

### 3.2. Isolation and Identification of Marine Endophytic Fungus

**Figure 5:**
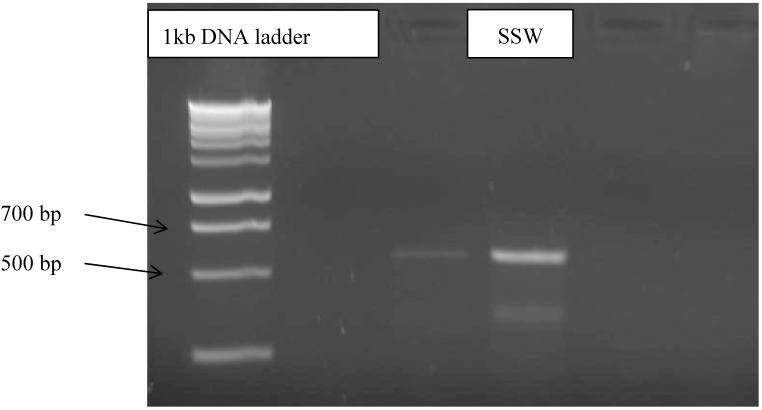
Gel electrophoresis of SSW strain PCR results.

Strain SSW was isolated from the marine algae sea weed collected at Pangkor Island, Malaysia, after surface sterilization following a published procedure [22]. The fungus grew on potato dextrose broth (PDB) The fungal in broth culture were freeze dry with liquid nitrogen followed by grinding with mortar and pestle until become powder. The DNA of the fungi was extracted using a kit purchased from Norgen Biotech^®^ (Thorold, Canada). The ITS region was amplified using universal primer, ITS 1 (TCCGTAGGTGAACCTGCGG) for the forward primer and ITS 4 (TCCTCCGCTTATTGATATGC) for the reverse primer [23]. The polymerase chain reaction (PCR) mixture consisted of 2.5 μL reaction buffer, 1 µL MgCl_2_, 0.2 μL taq polymerase, 1 μL ITS, 1 μL ITS 4, 0.5 μL dNTPs mix, and 15.8 μL distilled water was added to make the total volume of reaction 25 μL. The thermocycler programme was set to 1 min and 94 °C for the first cycle, followed by 30 s and 50 °C for the second cycle; the last cycle was done in 7 min and 72 °C. The PCR products were analyzed on 1.5% agarose gel for 35 min at 90 V. A 1-kb DNA ladder was used as size marker [24]. The results showed the ITS region amplified at 600–700 bp (Figure 5). The PCR product was purified using gel purification kit purchased from PROMEGA^®^ (Fitchburg, WI, USA) and sequenced by 1st BASE Sdn Bhd. The SSW strain sequence was blasted in National Centre for Biological Information (NCBI) Genebank. The blast result showed 99% homology with *Penicilium lapidosum* database sequence. 

### 3.3. Culture Conditions and Media Preparation

Endophyte *Penicilium lapidosum* SSW was grown on potato dextrose agar (PDA) at 28 °C and stored at 4 °C. Fermentation medium was prepared by adding glucose (10 g), peptone from meat (5 g), KH_2_PO_4_ (5 g), yeast extract granulated (5 g), sodium chloride (5 g), and glycerol (5 mL) into distilled water (1 L). The media were then transferred into 250 mL flasks containing 100 mL medium each and autoclaved at 120 °C. Inoculum of *Penicilium lapidosum* was prepared from seven-day old slant and allowed for 2 days fermentation.

### 3.4. Preliminary Screening Experiments

Twenty microorganisms including endophytes from our collection and ATCC strains were screened for microbial transformation by transferring mycelium from PDA slants in 100 mL conical flask containing 40 mL of medium at 28 °C with rotary shaking at 100 rpm. After two days of fermentation predinisolone (**1**, 2.0 mg) dissolved in acetone (0.25 mL) was added in each flask. The cultures were extracted with EtOAc after another 12 days of incubation. Culture controls consisted of microorganisms without substrate and substrate controls composed of sterile medium added with starting material without the microorganisms were incubated in the same conditions as described above. All extracts were analyzed by HPLC and chromatographic profiles examined for any additional peak(s). 

### 3.5. Preparative Fermentation

The preparative fermentation was carried out in two stages. Fragments of *Penicilium lapidosum* mycelium were transferred into two flasks under sterile conditions to prepare seed flasks. These inoculated flasks were kept on a rotary shaker for two days. The seed flasks were used to inoculate 18 flasks. After 2 days of shaking incubation at 128 rpm and 28 °C, predinisolone (**1**, 200 mg) was dissolved in acetone (5 mL) and evenly distributed among all flasks. The cultures were further incubated for 12 days in above conditions. The cultures were filtered and extracted three times with ethyl acetate. The solution was evaporated under reduced pressure (40 °C) to yield a crude residue (340 mg). 

### 3.6. Isolation of the Metabolites

The crude residue (340 mg) was first fractionated by column chromatography on silica gel (0.063–0.200 mm). Compound **2** eluted with ethyl acetate–methanol (98:2, v/v) with a minor impurity and was further purified by semi-preparative HPLC. The sample was dissolved in methanol (50 mg/mL) and filtered on a 0.45 µm of PTFE membrane. Aliquots of 10 µL were separated isocratically with a mobile phase made of purified water and acetonitrile (40:60) at a flow rate of 5 mL/min. The temperature of the column was maintained at 36 °C throughout the experiments. The separation was monitored at 220 nm. Pure biotransformed product **2** was found to elute at retention time 9.58 min.

### 3.7. Characterization of the Transformed Product 20β-Hydroxyprednisolone (**2**)

White amorphous solid (5.5 mg); mp 184–186 °C, ${\left[\alpha \right]}_{D}^{25}$ +138 (MeOH, *c* = 0.45); HRESI-MS 362.2134 [M + H]^+^ for C_21_H_30_O_5_ (calcd 362.2143); EI MS *m/z* (rel. int.%) 362 (M^+^, 6) 346 (11), 344 ([M-H_2_O]^+^, 30), 329 (21), 300 (22), 243 (16), 189 (14), 187 (13), 176 (24), 161 (21), 149 (26), 135 (22), 121 (25), 91 (87), 81 (23), 69 (11), 67 (23), 57 (41); IR ν_max_ 3461, 2930, 1716, 1658 and 1615 cm^−1^; ^1^H and ^13^C-NMR data in Table 2 and Table 3.

### 3.8. Theoretical Details

The geometry optimization of the ground-state (GS) of 20β-hydroxypredinisolone and 20α-hydroxypredinisolone have been carried out using density functional theory (DFT) at the B3LYP/6–31+G(d,p) level of theory [25]. The frequency analyses were performed at the same level of theory. The absence of imaginary frequencies confirms the ground states minima. Excited singlet state (ES) energies were calculated from the optimized geometries using TD-DFT at the B3LYP/6–31+G(d,p) level [26,27]. The allowed vertical electronic excitation energies were thus obtained, which consequently give the absorption energies in the UV/vis range with their CI (configuration interaction) description as well as their oscillator strength (*f* > 0 for allowed transition). In correspondence with the apparent absorption bands, chiral compounds show intense CD bands or Cotton effects (CE). A positive CE corresponding to the (*R*) enantiomer and a negative CE to the (*S*) one. The predicted ^1^H- and ^13^C-NMR magnetic isotropic shielding tensors (σ) were determined by standard Gauge-Independent Atomic Orbital approach (GIAO) [28], at the same level of theory (B3LYP/6–31+G(d,p). The isotropic shielding values were used to calculate the isotropic chemical shifts δ with respect to tetramethylsilane (TMS). δ_iso_(X) = σ_TMS_(X) − σ_iso_(X), where δiso is isotropic chemical shift and σ_iso_ isotropic shielding constant. The predicted chemical shifts were obtained using the equation δ_exp_ = aδ_cal_ + b, where δ_cal_ = δ_iso_.

The theoretical calculations were performed in gas and in solvent. The solvent effects were taken into account implicitly by using the polarizable continuum model (PCM) [21]. In PCM, the solute is embedded into a shape-adapted cavity surrounded by solvent, which implicitly described as a dielectric continuum characterized by its dielectric constant. CDCl_3_ (ε = 4.7113) was used for ^1^H and ^13^C-RNMR chemical shift predictions, while CH_3_CN (ε = 35.688) was used in TD-DFT calculations for UV/vis and CD values predictions. In TD-DFT calculations, the PCM correctly models the major solvent effects such as electrostatic effects of the medium as long as no specific solute-solvent interaction is considered like hydrogen bond interactions, dipole-dipole interactions, or induced dipole-dipole interactions between solvent and solute [29]. In a recent study, Liu *et al.* showed that dipole-dipole interactions between coumarin and solvent molecules lead to larger red shift [30]. DFT and TD-DFT calculations were performed using Gaussian09 package [31].

## 4. Conclusions

Predinisolone (**1**) was biotransformed by marine endophytic fungus *Penicilium lapidosum* isolated from a marine alga. It was stereoselectively reduced to 20β-hydroxyprednisolone (**2**). Although this compound is known, no convincing arguments were available from the literature to establish unambiguously its stereochemistry at C20. The use of DFT and TD-DFT calculations led us to determine that most convincing and discriminating spectroscopic data were the ^3^*J*_HH_ coupling constants for H_2_O. The 20*S* configuration would result in experimental values of 3 and 8.5–9 Hz, while these values would drop to 1 and 2 Hz (absolute values) for the 20*R* configuration. This work illustrates the power of quantum chemistry methods to solve problems that experimentalists may face in the course of their work. 
